# Haemolytic anaemia due to severe melody pulmonary valve stenosis caused by *Histoplasma capsulatum* infective endocarditis: a case report

**DOI:** 10.1093/ehjcr/ytag380

**Published:** 2026-05-30

**Authors:** Brock J Gilsdorf, Charles A Tharp, Joseph M Burke

**Affiliations:** Internal Medicine Residency Program, Department of Medicine, University of Colorado School of Medicine, 13001 E. 17th Place, Aurora, CO 80045, USA; Division of Cardiology, Denver Health Medical Center, 777 Bannock Street, Denver, CO 80204, USA; Division of Cardiology, Denver Health Medical Center, 777 Bannock Street, Denver, CO 80204, USA

**Keywords:** Endocarditis, Haemolysis, Histoplasma, Pulmonary stenosis, Melody, Case report

## Abstract

**Background:**

Isolated pulmonary valve infective endocarditis (IE) is exceedingly rare, accounting for <2% of IE cases. Haemolysis due to right-sided prosthetic valve dysfunction is also poorly described.

**Case summary:**

We present a patient with tetralogy of Fallot and a Melody pulmonary valve replacement who presented with dyspnoea on exertion and scleral icterus found to have isolated pulmonary valve IE caused by *Histoplasma capsulatum*, resulting in severe valve stenosis and intravascular hemolysis. Diagnosis was established through exclusion of other causes of hemolysis, transthoracic echocardiography showing severe prosthetic stenosis, and infectious evaluation identifying *H. capsulatum*, confirmed by histopathology of the explanted valve. The patient was treated with antifungal therapy followed by surgical pulmonary valve replacement, leading to clinical improvement.

**Discussion:**

Fungal endocarditis due to *Histoplasma* is rare, particularly involving the pulmonary valve. Haemolysis from right-sided valve dysfunction is an extraordinarily rare clinical presentation. This case highlights the importance of considering prosthetic valve IE in unexplained haemolysis and using multimodal diagnostic strategies.

Learning points

- When more common causes of intravascular haemolysis are ruled out, consideration of right-sided valvular dysfunction should be considered, particularly in patients with prosthetic valves.
- Diagnosis of pulmonary valve infective endocarditis is rare, as is *Histoplasma capsulatum*, which requires a thorough infectious workup to establish the diagnosis.

## Introduction

Isolated pulmonary valve infective endocarditis (IE) accounts for <2% of all IE cases. Major risk factors include the presence of a prosthetic valve, intravenous drug use, congenital heart disease, and alcohol use disorder.^1,2,3^ The rarity of pulmonary valve involvement is thought to reflect lower right-sided pressures, reduced oxygen content, and a lower prevalence of congenital or acquired pulmonary valvular abnormalities compared with left-sided valves.^1,2^

Gram-positive staphylococci, streptococci, and enterococci account for approximately 80%–90% of IE cases, while fungal aetiologies are rare.^4,5^  *Histoplasma capsulatum* is an uncommon cause of IE and has been reported predominantly in native and prosthetic left-sided valves. From 1940 to 2020, only 36 cases of native valve and 25 cases of prosthetic valve endocarditis due to *H. capsulatum* were reported, with the majority involving the aortic valve and no reported cases of prosthetic pulmonary valve endocarditis.^6^

Haemolysis is a well-known complication of left-sided valve repairs and support devices.^7^ However, haemolysis from right-sided valve dysfunction is rare. The original Melody investigational device exemption (IDE) trial had no cases of post-implantation haemolysis, and no evidence of stenosis at 10 years with only 2% per patient-year risk of IE.^8^ To our knowledge, only one case report of post-implantation intravascular haemolysis due to the Melody valve has been reported.^9^

We present a rare case of an adult with tetralogy of Fallot (TOF) and prior Melody pulmonary valve replacement who developed isolated pulmonary valve infective endocarditis due to *H. capsulatum*, resulting in severe pulmonic valve stenosis and intravascular haemolysis.

## Summary figure

**Figure:**
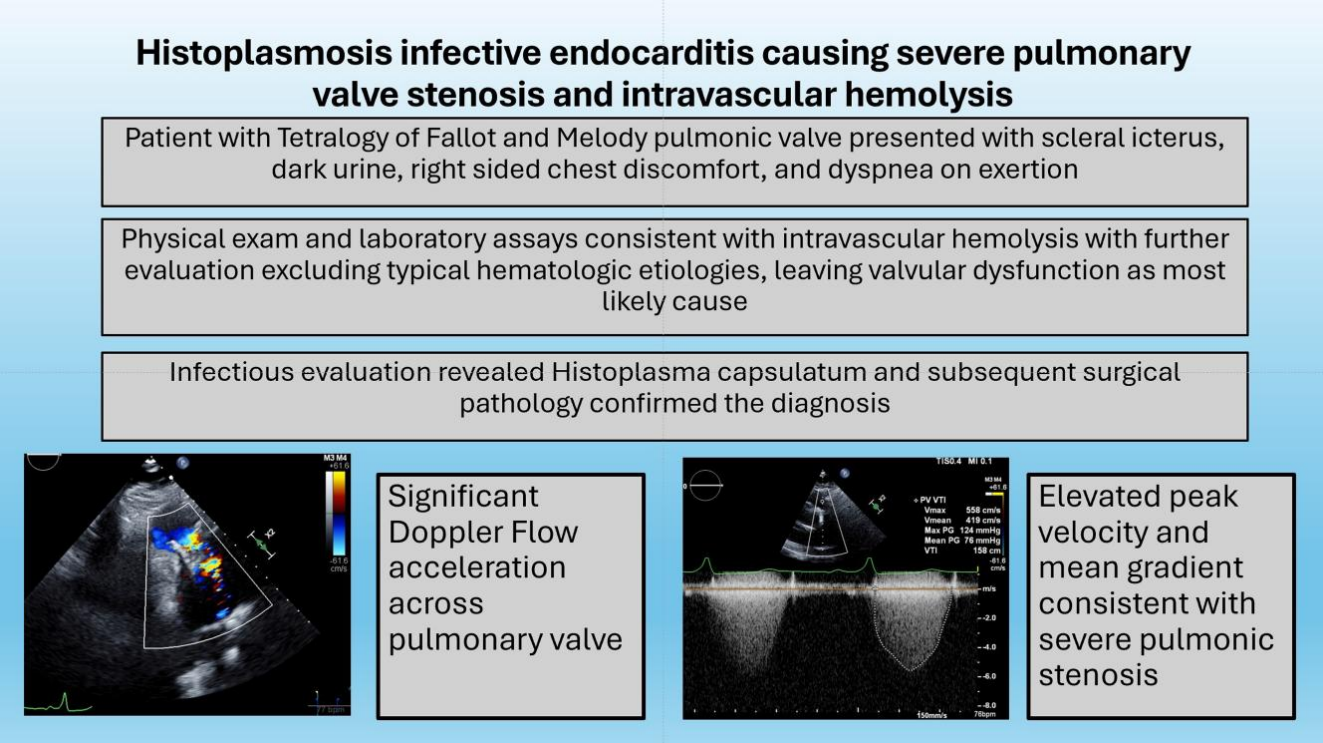


## Case presentation

A 24-year-old female with a history of tetralogy of Fallot (TOF) presented with 1 week of scleral icterus and dark urine. She also reported right-sided chest discomfort exacerbated by stretching and two weeks of progressively decreased exercise tolerance, now unable to run on a treadmill or climb a single flight of stairs. She denied fever, chills, palpitations, cough, congestion, rhinorrhoea, or dysuria.

The patient had a notable travel history. Ten months prior to presentation, she travelled across Thailand, Vietnam, the Philippines, Indonesia, Singapore, and South Korea where she was appropriately vaccinated prior to travel and only experienced a self-limited diarrheal illness during her travels. Upon returning, she received outpatient workup for diarrhoea including an unremarkable stool ova and parasite (O&P) and faecal calprotectin.

On presentation, vital signs were temperature 37.1°C, heart rate 85 beats per minute, respiratory rate 20 breaths per minute, blood pressure 117/69 mmHg, and oxygen saturation 97% on room air. Physical examination was notable for scleral icterus and a harsh systolic murmur. Electrocardiography demonstrated sinus rhythm without bundle branch block or T-wave abnormalities.

**Figure 1:**
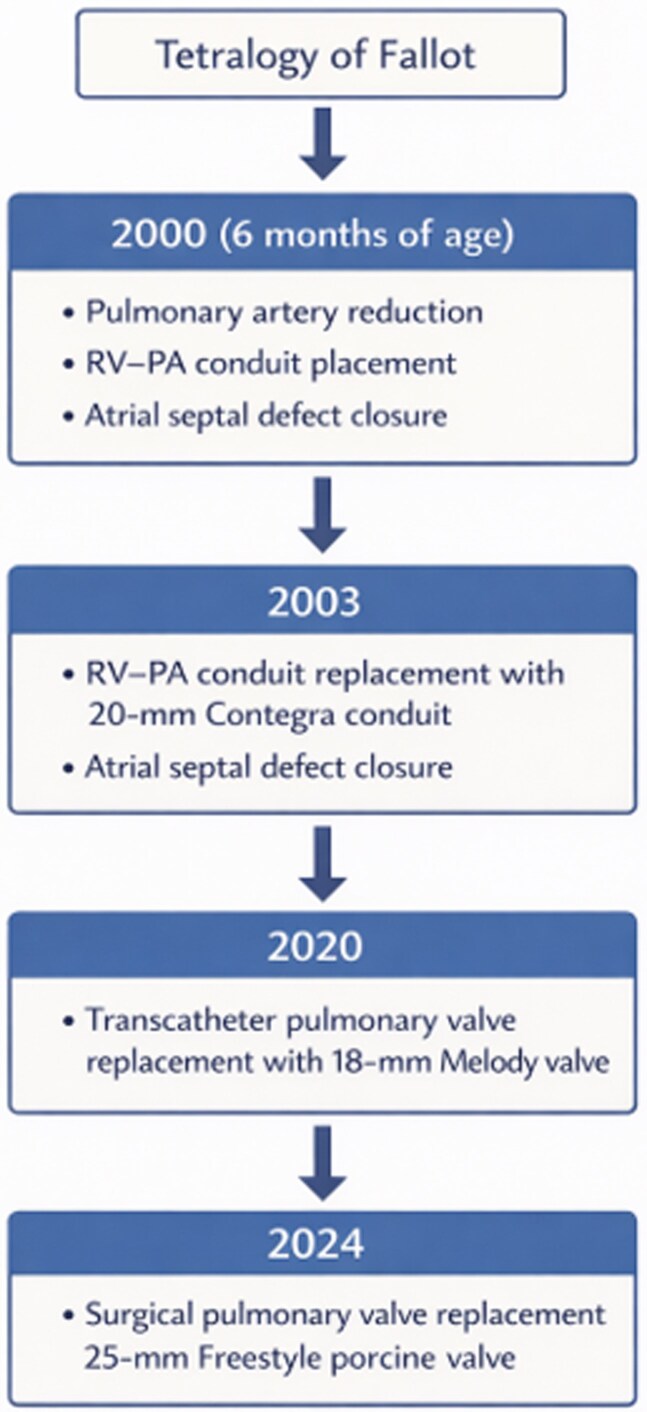
Timeline depicting the patient’s congenital cardiac interventions from infancy through adulthood, including pulmonary artery reduction with right ventricle-to-pulmonary artery conduit placement and atrial septal defect closure, subsequent RV–PA conduit replacement with a 20-mm Contegra conduit, transcatheter pulmonary valve replacement with an 18-mm melody valve, and most recent surgical pulmonary valve replacement with a 25-mm Freestyle porcine valve.

The patient had a congenital diagnosis of TOF with an absent pulmonary valve and underwent complete repair at 6 months of age, including pulmonary artery reduction, right ventricle-to-pulmonary artery (RV–PA) conduit placement, and atrial septal defect (ASD) closure (*Figure 1*). At 3 years of age, she underwent repeat ASD closure and RV–PA conduit replacement with a 20-mm Contegra bovine jugular vein graft. At 20 years of age, the patient underwent transcatheter pulmonary valve replacement with an 18-mm Melody valve. Her only chronic medication was aspirin. A routine transthoracic echocardiogram performed 4 months prior to presentation demonstrated normal valve gradients and function.

Laboratory evaluation revealed a white blood cell count of 6.1 k/uL, haemoglobin of 10.5 g/dL, platelets of 160 k/uL, and peripheral smear with 1+ schistocytes. Electrolytes and kidney function were within normal limits. Urinalysis showed 3+ blood, negative nitrites, trace leukocytes, and 11–20 red blood cells.

Inflammatory markers were normal with an erythrocyte sedimentation rate (ESR) < 1 mm/h and C-reactive protein (CRP) < 4 mg/L. Additional testing demonstrated a total bilirubin of 4.3 mg/dL, indirect bilirubin of 3.3 mg/dL, lactate dehydrogenase of 2316 U/L, haptoglobin <7 mg/dL, and reticulocyte index of 3.56%, consistent with haemolysis. High-sensitivity troponin I was 17 ng/L, and NT-proBNP was 3156 pg/ml. Coagulation studies were normal with an international normalized ratio (INR) of 1.07. Direct antiglobulin test (DAT), HIV, and hepatitis C were all negative. Stool PCR and ova and parasite evaluation were unremarkable. Finally, testing for paroxysmal nocturnal haemoglobinuria (PNH) and glucose-6-phosphate dehydrogenase (G6PD) deficiency was negative.

**Figure 2:**
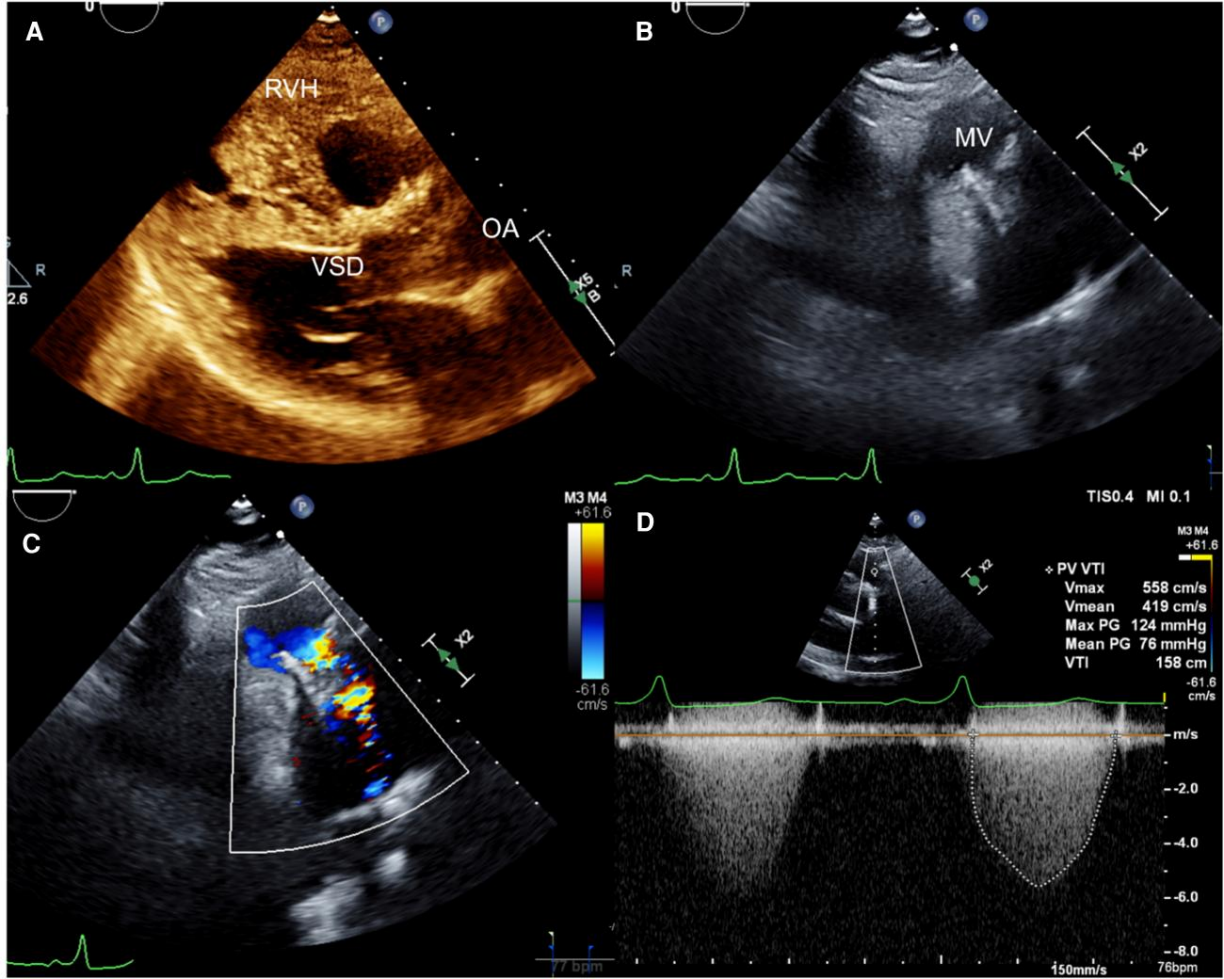
Transthoracic echocardiography at clinical presentation. (*A*) Parasternal long-axis view demonstrating findings consistent with tetralogy of Fallot including right ventricular hypertrophy, repaired ventricular septal defect, and overriding aorta. (*B*) Parasternal short-axis view of the pulmonic valve with prosthetic stent-mounted valve replacement. (*C*) Parasternal short-axis view of the pulmonic valve with colour Doppler demonstrating significant flow acceleration associated with prosthetic stenosis. (*D*) Continuous wave spectral Doppler across the prosthetic pulmonic valve in parasternal short axis demonstrating elevated peak velocity and mean gradient consistent with severe pulmonic stenosis.

Transthoracic echocardiography demonstrated a transcatheter bioprosthetic valve in the pulmonic position with a transpulmonic velocity of 6.0 m/s, corresponding to a peak gradient of 124 mmHg and a mean gradient of 79 mmHg, consistent with severe prosthetic pulmonic valve stenosis (*Figure 2*).

Haematology was consulted regarding patient’s haemolytic anaemia. Microangiopathic haemolytic anaemia was considered unlikely due to normal platelets. Autoimmune aetiologies were deemed less likely due to normal CRP, ESR, and negative DAT. Disseminated intravascular coagulation (DIC) was unlikely with normal INR. The patient had a negative PNH and G6PD deficiency evaluation. Given the exclusion of alternative causes, the patient’s severe haemolysis was attributed to markedly elevated transvalvular gradients from severe prosthetic pulmonic valve stenosis.

Further evaluation was pursued to determine the aetiology of her pulmonic valve stenosis, as it was functioning properly only 4 months prior. A broad infectious workup revealed elevated *Histoplasma* antigen levels (0.98 ng/ml in serum and 0.34 ng/ml in urine). Serologic testing was positive, with *Histoplasma* IgM of 57.9 ELISA units (EU) and IgG >80 EU by gel diffusion, as well as yeast antibody titres of 1:32 and mycelial antibody titres of 1:64 by complement fixation. Karius testing, a blood test that uses next-generation sequencing to identify pathogens, returned positive for *H. capsulatum*. Blood and fungal cultures remained negative throughout.

She was started on 6 weeks of amphotericin B (3 mg/kg q24 h) and 1 year of itraconazole therapy. After 1 week of antifungal treatment, she underwent open heart surgery which demonstrated a heavily calcified valve, with three-quarters of the valve area being closed off. She had a pulmonary valve replacement with a 25-mm Freestyle porcine aortic root and augmentation of the right ventricular outflow tract with a bovine pericardial patch. A sample obtained from the removed valve noted hyphal elements. Repeat serum *Histoplasma* antigen testing performed three days after valve replacement was negative.

## Discussion

Intravascular haemolysis due to left-sided valvular dysfunction is well described, but to our knowledge, haemolysis from isolated pulmonary valve endocarditis and resulting stenosis has not been reported, nor has a case of prosthetic pulmonary valve *H. capsulatum* endocarditis been reported previously. This case describes prosthetic pulmonary valve stenosis due to *H. capsulatum* endocarditis causing clinically significant haemolysis in a patient with repaired tetralogy of Fallot and prior transcatheter pulmonary valve replacement.

This case underscores the importance of considering right-sided valvular dysfunction in patients with unexplained haemolysis. After common aetiologies were excluded, cardiac imaging revealed severe pulmonary valve stenosis with markedly elevated transvalvular gradients, implicating prosthetic valve dysfunction as the underlying cause. Although the Melody IDE trial and subsequent studies have reported only one case of post-implantation haemolysis, our case demonstrates that severe obstruction of a bioprosthetic right-sided valve can generate sufficient shear stress to cause clinically significant haemolysis. Intraoperative identification of approximately 75% valve occlusion further supported this mechanism.

This case also illustrates the diagnostic challenges of IE, especially if due to *H. capsulatum* which is a fungus that is an exceedingly uncommon cause of IE with only one prior case report involving a pulmonary valve. Our patient’s presentation lacked typical infectious signs, including fever, leucocytosis, or positive blood cultures. The abrupt onset of severe stenosis in a previously functional valve prompted further evaluation, ultimately confirming *Histoplasma* through antigen testing, next-generation sequencing, and surgical tissue analysis.

In repaired tetralogy of Fallot, pulmonary valve function requires longitudinal assessment because chronic pulmonary regurgitation commonly develops over time, in contrast to the infective valvular stenosis seen in our case.^10^ While echocardiography remains useful for evaluating pulmonary valve function after repair, cardiac magnetic resonance is the preferred modality for quantifying pulmonary regurgitation and right ventricular size and function, thereby helping determine the timing of re-intervention.^10,11^

Our case highlights that although chronic pulmonary regurgitation is the more typical long-term valvular complication after tetralogy of Fallot repair, abrupt prosthetic pulmonary valve stenosis should prompt evaluation for superimposed pathology, including infective endocarditis. In summary, this case reports the first known instance of clinically significant haemolysis resulting from right-sided prosthetic pulmonary valve dysfunction and the first reported case of *H. capsulatum* prosthetic pulmonary valve endocarditis.

## Data Availability

Data from this case report is available on reasonable request. Please contact the corresponding author.

## Lead author biography

Brock J. Gilsdorf, MD, is currently a third year internal medical resident at the University of Colorado School of Medicine. Dr. Gilsdorf completed his medical degree at the University of Wisconsin School of Medicine and Public Health. Dr. Gilsdorf was applying to a cardiovascular disease fellowship at the time of manuscript submission and, during the revision process, matched to the University of Wisconsin Hospital and Clinics Cardiovascular Fellowship.

## Notes

**Consent:** Informed consent was obtained from the patient for the preparation of this manuscript in compliance with COPE guidelines.
